# Inflammation- and Metastasis-Related Proteins Expression Changes in Early Stages in Tumor and Non-Tumor Adjacent Tissues of Colorectal Cancer Samples

**DOI:** 10.3390/cancers14184487

**Published:** 2022-09-16

**Authors:** Marina Alorda-Clara, Margalida Torrens-Mas, Reyniel Hernández-López, Javier M. Ibarra de la Rosa, Esther Falcó, Teresa Fernández, Maria Margarita Company, Jorge Sastre-Serra, Jordi Oliver, Daniel Gabriel Pons, Pilar Roca

**Affiliations:** 1 Grupo Multidisciplinar de Oncología Traslacional, Institut Universitari d’Investigació en Ciències de la Salut (IUNICS), Universitat de les Illes Balears, 07122 Palma de Mallorca, Spain; 2 Instituto de Investigación Sanitaria Illes Balears (IdISBa), Hospital Universitario Son Espases, Edificio S, 07120 Palma de Mallorca, Spain; 3 Translational Research in Aging and Longevity (TRIAL) Group, Instituto de Investigación Sanitaria Illes Balears (IdISBa), 07120 Palma de Mallorca, Spain; 4 Pathological Anatomy, Hospital Universitari Son Llàtzer, 07198 Palma de Mallorca, Spain; 5 Medical Oncology Service, Hospital Universitari Son Llàtzer, 07198 Palma de Mallorca, Spain; 6 Pathological Anatomy, Clinica Rotger, 07012 Palma de Mallorca, Spain; 7 Ciber Fisiopatología Obesidad y Nutrición (CB06/03), Instituto Salud Carlos III, 28029 Madrid, Spain

**Keywords:** inflammation, metastasis, epithelial-mesenchymal transition, tumor tissue, non-tumor adjacent tissue, colorectal cancer

## Abstract

**Simple Summary:**

Non-tumor adjacent tissue plays a key role in colorectal cancer development, as well as chronic inflammation, but their role has not yet been dilucidated. In addition, inflammation is a process which is related to epithelial-mesenchymal transition and metastasis, but their changes across the different colorectal cancer stages are not fully studied. Understanding how these processes participate in all colorectal cancer phases can be key to a better understanding of the disease.

**Abstract:**

Chronic inflammation can induce malignant cell transformation, having an important role in all colorectal cancer (CRC) phases. Non-tumor adjacent tissue plays an important role in tumor progression, but its implication in CRC has not yet been fully elucidated. The aim was to analyze the expression of inflammatory, epithelial-mesenchymal transition (EMT), and metastasis-related proteins in both tumor and non-tumor adjacent tissues from CRC patients by western blot. Tumor tissue presented an increase in metastasis and EMT-related proteins compared to non-tumor adjacent tissue, especially in stage II. Tumor tissue stage II also presented an increase in inflammatory-related proteins compared to other stages, which was also seen in non-tumor adjacent tissue stage II. Additionally, the relapse-free survival study of Vimentin and VEGF-B expression levels in stage II patients showed that the higher the expression levels of each protein, the lower 10-year relapse-free survival. These could suggest that some metastasis-related signalling pathways may be activated in stage II in tumor tissue, accompanied by an increase in inflammation. Furthermore, non-tumor adjacent tissue presented an increase of the inflammatory status that could be the basis for future tumor progression. In conclusion, these proteins could be useful as biomarkers of diagnosis for CRC at early stages.

## 1. Introduction

Colorectal cancer (CRC) is the third most common cancer worldwide, being the second cause of cancer death [1]. The American Cancer Society has established the 5-year survival rates for each CRC stage: the 5-year survival rate for localized tumors is 91%; 72% for cancer that has spread to surrounding tissues or lymph nodes; and finally, 14% for cancer that has spread to distant parts of the body [2]. These data highlight the importance of early detection of this disease. CRC can be classified attending to the TNM staging system and, depending on the tumor extension, can be divided into four stages, numbered from I to IV, with stages I to III being non-metastatic stages, and stage IV the metastatic stage. In stage I, the tumor grows through the mucosa to the muscular layer without dissemination to lymphatic nodes. In stage II, the tumor grows through the muscular layer to nearby structures without dissemination to lymphatic nodes. In stage III, the tumor spreads to nearby organs and the lymphatic nodes, and finally, in stage IV, the tumor spreads to other parts of the body through lymphatic nodes and blood vessels [3].

Despite inflammation being a physiological process, when it is upregulated and becomes chronic, it can induce malignant cell transformation in the surrounding tissue [4]. Inflammation, one of the cancer hallmarks [5], participates in all CRC phases since cytokines are released in response to tissue damage, so innate immune system cells are recruited and release more cytokines [6]. This positive feedback leads to cell survival, immunosuppression, tumor growth, proliferation, differentiation, angiogenesis, epithelial-mesenchymal transition (EMT), and metastasis [4,7,8,9]. EMT is a biological process where epithelial cells acquire a mesenchymal phenotype and the capability to migrate, invade and metastasize [10]. With the contribution of cytokines, extracellular matrix degradation, invasion, tumor cell evasion, and dissemination, can be possible and lead to metastasis [6].

Non-tumor adjacent tissue plays an important role in tumor progression [8,11]. Oxidative stress-related proteins were analyzed in tumor and non-tumor adjacent tissue from advanced stages (III and IV), and the non-tumor adjacent tissue presented the highest differences [8]. Furthermore, when this family of proteins was analyzed in non-metastatic stages (I, II, and III), the non-tumor adjacent tissue showed higher levels of antioxidant enzymes, although the tumor tissue presented higher levels of manganese superoxide dismutase (SOD2) and acetylated SOD2. On the other hand, the major differences were found between stages I and II in both tissues [11]. These results indicate that antioxidant enzymes play a crucial role in both tumor and non-tumor adjacent tissues in CRC [8,11].

The aim of this study was to analyze the expression of inflammatory, EMT, and metastasis-related proteins in both tumor and non-tumor adjacent tissues in all stages (I, II, III, IV) from CRC patients’ samples, to gain a better understanding of the relationship between those biological processes.

## 2. Materials and Methods

### 2.1. Reagents

Routine reagents were purchased in Panreac (Barcelona), Sigma-Aldrich (St. Louis, MO, USA), and Bio-Rad Laboratories (Hercules, CA, USA).

### 2.2. Patients and Tissue Samples

To carry out the study, a total of 38 patients were included. Patients of both sexes without comorbidity were included. Furthermore, patients did not receive chemotherapy or radiotherapy before surgery and had signed an informed consent according to “World Medical Association Declaration of Helsinki” when medical research involves humans.

Tumor tissues and non-tumor adjacent tissues were recollected from *Biobanc de Son Llàtzer*, for 5 years (2005–2010), and maintained at −80 °C until their processing to carry out the study. Patient samples were divided into stages (I, II, III, and IV) and into tissue types (tumor tissue and non-tumor adjacent tissue). A total of 8 samples were included in stage I (5 males and 3 females), 10 samples were included in stage II (5 males and 5 females), 8 samples were included in stage III (2 males and 6 females), and 12 samples were included in stage IV (9 males and 3 females). The age average was 70.8 years and SD was 11.9 (male age average was 72.1 ± 11.6 and range 51-95; female age average was 69.1 ± 12.5 and range 50–88). Clinicopathological features of patient samples included in the study are shown in Table S1. Both tissues were histologically examined by a pathologist and classified by TNM staging system.

### 2.3. Tissue Homogenization and Protein Quantification

Frozen tumor and non-tumor adjacent tissues were homogenized in a proportion 1:10 (*w*/*v*) in homogenization buffer (Tris 20 mM pH 7.4, Saccharose 250 mM, EGTA 2 mM and KCl 40 mM) with polytron (T10 basic, IKA-Werke 6 mbH, Staufen, Germany). After that, homogenates were sonicated with Vibra Cell Ultrasonic Processor 75185 on ice in two cycles of 25 W for 5 s with an interval of 85 s between pulses and 40% of amplitude. Finally, homogenates were centrifugated at 600 xg for 10 min at 4 °C and supernatant was recovered. Protease and phosphatase inhibitors (PMSF 1 mM, NaF 1 mM, Pepstatin 10 µM, Leupeptin 10 µM and Na_3_VO_4_ 1 mM) were added to the supernatant. Protein levels were quantified by Bradford method [12].

### 2.4. Western Blotting

For all SDS-PAGE carried out, 25 µg of total protein was loaded. Electrophoresis for protein separation was conducted on 8%, 10%, and 12% acrylamide/bisacrylamide (30/1) gels. A semi-dry electrotransfer was perfomed to transfer proteins into a 0.2 *µ*m nitrocellulose membrane (Bio-Rad Laboratories, Hercules, CA, USA) using a trans-blot turbo transfer system (Bio-Rad Laboratories, Hercules, CA, USA). After electrotransfer, membranes were incubated with Ponceau staining for equal sample loading validation. Then, membranes were blocked with 5% bovine serum albumin (BSA) or non-fat powdered milk (for phosphorylated and non-phosphorylated proteins, respectively) in Tris Buffer Saline 0.05% Tween 20 pH 7.6 (TBS-Tween) for 1 h at room temperature and agitation. After TBS-Tween washes, membranes were incubated with primary antibody (5% free fatty acids BSA and 0.05% sodium azide in TBS-Tween) over night at 4 °C and agitation. Primary antibodies used and their respective dilutions were: inhibitor of kappa B phosphorylated (pIκB) 1:1000 (Cell Signaling, cs2859), inhibitor of kappa B (IκB) 1:1000 (Cell Signaling, cs4814), interferon gamma (IFNγ) 1:1000 (Abcam, 9657), peroxisome proliferator activated receptor gamma (PPARγ) 1:200 (Santa Cruz Biotechnology, sc-7196), interleukin 4 receptor (IL-4R) 1:1000 (Santa Cruz Biotechnology, sc-28361), cyclooxygenase 2 (COX2) 1:1000 (Boster Biological, PA1211), heparanase 1:500 (Abcam, 232817), matrix metalloproteinase 9 (MMP9) 1:1000 (Cell Signaling, cs13667), vimentin 1:500 (Santa Cruz Biotechnology, sc-373717), E-cadherin 1:200 (Santa Cruz Biotechnology, sc-8426), N-cadherin 1:100 (Santa Cruz Biotechnology, sc-393933) and vascular endothelial growth factor B (VEGF-B) 1:500 (Cell Signaling, cs2463). After that, TBS-Tween washes were made. Finally, membranes were incubated with horseradish peroxidase-conjugated secondary antibody (2% BSA or non-fat powdered milk in TBS-Tween) for 1 h at room temperature and agitated. Secondary antibodies used and their respective dilutions were: anti-rabbit 1:10,000 (Sigma, A9169) and anti-mouse 1:10,000 (Sigma, A9044). After incubation, membranes were washed and Inmun-Star© Western Chemilumeniscence kit Western blotting detection systems (Bio-Rad Laboratories, Hercules, CA, USA) was used to detect the immunoreactivity. Chemidoc XRS densitometer (Bio-Rad Laboratories, Hercules, CA, USA) was used to acquire chemiluminescent signal for pIκB, IκB, IFNγ, PPARγ, IL-4R, COX2, heparinase and MMP9, and, finally, the Quantity One Software (Bio-Rad Laboratories, Hercules, CA, USA) was used to analyze the results. For vimentin, VEGF-B, E-cadherin, and N-cadherin chemiluminescent signal was detected with the ImageQuant LAS 4000 mini Biomolecular Imager (GE Healthcare, Spain). A sample consisting of a mixture of stages I-IV tumor and non-tumor adjacent tissues was loaded as a control to allow comparison between gels, as previously published [8,11,13].

### 2.5. Kaplan–Meier Survival Curves

Relapse-free survival was assessed for 585 colon cancer patients from GSE39582 dataset [14,15]. The Statistical Program for the Social Sciences software for windows (SPSS, version 24.0; SPSS Inc., Chicago, IL, USA) was used to perform survival curves using Kaplan–Meier curves survival analysis and the Log Rank test for statistical significance, which was set at *p* < 0.05. Patients were divided into stages (I, II, III, and IV) and low or high Vimentin or VEGF-B expression levels.

### 2.6. Statistical Analysis

SPSS was used to perform all statistical analyses. Two-way ANOVA was used to analyze differences between tissues and stages. Student’s *t*-test was used to analyze differences between stages or tissues in proteins with an interactive effect found by the two-way ANOVA, with minimal statistical significance at *p* < 0.05. All results are presented as box-plot graphs and relativized to the mean of non-tumor adjacent tissue stage I samples, which was set as 100%.

## 3. Results

### 3.1. Epithelium-Mesenchymal Transition Proteins Expression Levels

**Figure 1:**
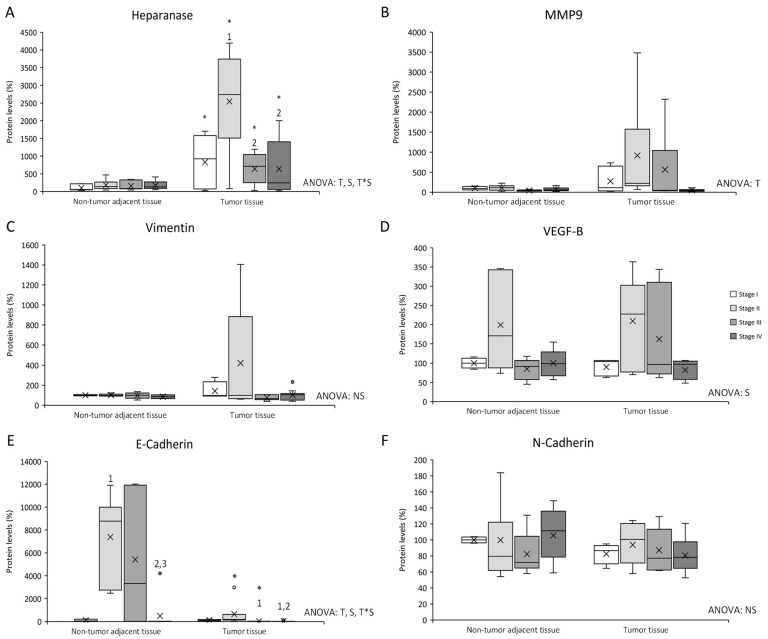
Changes in metastasis–related protein expression levels determined by Western blot: (**A**) Heparanase; (**B**) MMP9; (**C**) Vimentin; (**D**) VEGF-B; (**E**) E-cadherin; and (**F**) N-cadherin. Results are presented as box-plot graphs and relativized to the mean of non-tumor adjacent tissue stage I samples, which was set as 100%; the x in the box represents the mean. **NS:** non-significant differences, **T:** Significant differences between tissue types, **S:** significant differences between stages in the same tissue, T*S: interactive effect (ANOVA; *p* < 0.05). * Significant differences between non-tumor adjacent tissue and tumor tissue at the same stage, **1:** significant differences compared to stage I at the same tissue, **2:** significant differences compared to stage II at the same tissue, **3:** significant differences compared to stage III at the same tissue (Student’s *t*-test; *p* < 0.05). Original blots are presented in Supplementary Information File S1.

Heparanase, MMP9, vimentin, and VEGF-B protein levels, were studied in both tumor and non-tumor adjacent tissues of all stages from CRC patients (see Figure 1 and Figure 4).

Heparanase levels (Figure 1A) presented differences between non-tumor adjacent tissue and tumor tissue, differences between stages in the same tissue, and an interactive effect between tissue and stage. Tumor tissue showed an increase in heparanase levels compared to non-tumor adjacent tissue, in all four stages. Furthermore, tumor tissue stage II heparanase levels were higher than tumor tissue stages I, III, and IV.

MMP9 levels (Figure 1B) exhibited differences between non-tumor adjacent tissue and tumor tissue, with tumor tissue MMP9 levels higher than in non-tumor adjacent tissue.

Vimentin levels (Figure 1C) showed no statistically significant differences between tissues or stages.

VEGF-B levels (Figure 1D) presented differences between stages, with stage II VEGF-B levels higher than the other three stages.

E-cadherin levels (Figure 1E) presented differences between non-tumor adjacent tissue and tumor tissue, differences between stages in the same tissue, and an interactive effect between tissue and stage. Tumor tissue stages II and III showed a decrease in E-cadherin levels compared to non-tumor adjacent tissue. Furthermore, non-tumor adjacent tissue stage II E-cadherin levels were higher than non-tumor tissue stage I. In addition, stage IV E-cadherin levels were lower than stages II and III. Tumor tissue stages III and IV E-cadherin levels were lower than stage I, in addition to a decrease of stage IV when it was compared to stage II.

N-cadherin levels (Figure 1F) showed no statistically significant differences between tissues or stages.

### 3.2. Inflammatory-Related Proteins Expression Levels

**Figure 2:**
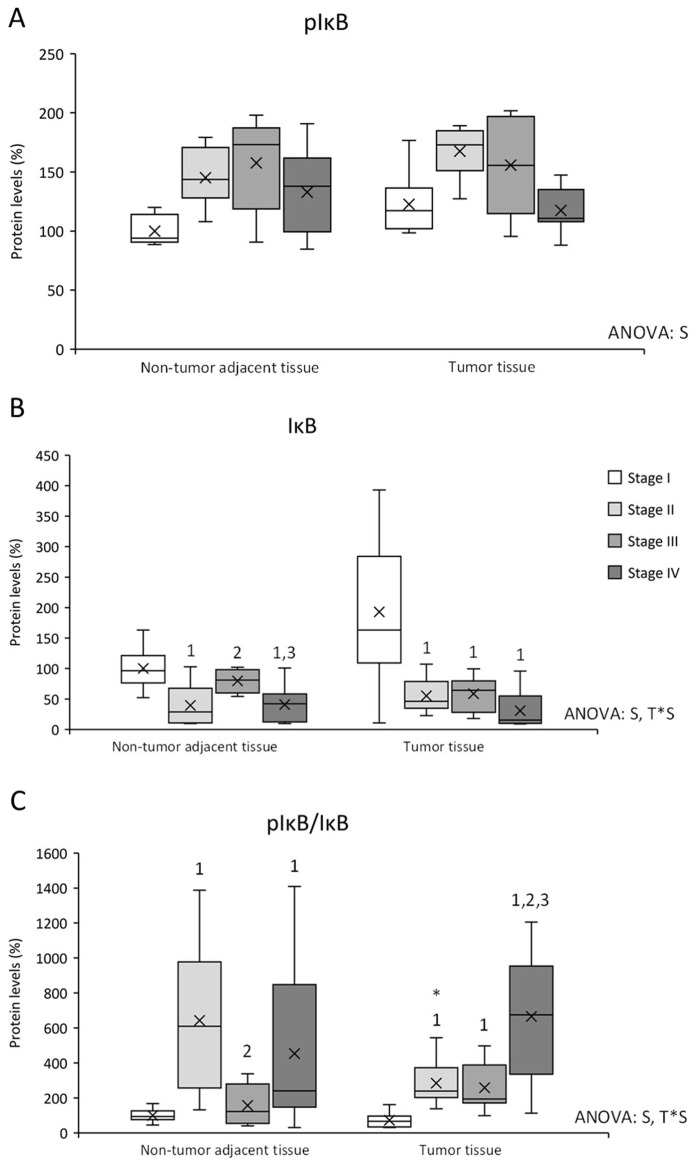
Changes in inflammation-related protein expression levels determined by Western blot: (**A**) pIκB; (**B**) IκB; and (**C**) pIκB/IκB ratio. Results are presented as box-plot graphs and relativized to the mean of non-tumor adjacent tissue stage I samples, which was set as 100%; the x in the box represents the mean. **NS:** non-significant differences, **T:** significant differences between tissue types, **S:** significant differences between stages in the same tissue, T*S: interactive effect (ANOVA; *p* < 0.05). * Significant differences between non-tumor adjacent tissue and tumor tissue at the same stage, **1:** significant differences compared to stage I at the same tissue, **2:** significant differences compared to stage II at the same tissue, **3:** significant differences compared to stage III at the same tissue (Student’s *t*-test; *p* < 0.05). Original blots are presented in Supplementary Information File S1.

**Figure 3:**
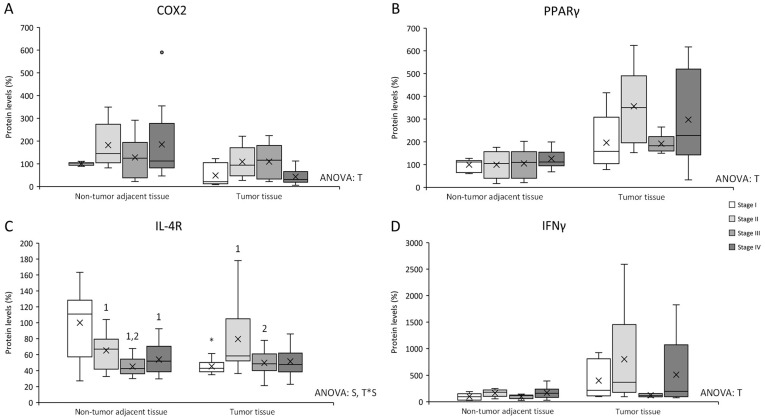
Changes in inflammation–related protein expression levels determined by Western blot: (**A**) COX2; (**B**) PPARγ; (**C**) IL-4R; and (**D**) IFNγ. Results are presented as box-plot graphs and relativized to the mean of non-tumor adjacent tissue stage I samples, which was set as 100%; the x in the box represents the mean. **NS:** non-significant differences, **T:** significant differences between tissue types, **S:** significant differences between stages in the same tissue, T*S: interactive effect (ANOVA; *p* < 0.05). * Significant difference between non-tumor adjacent tissue and tumor tissue at the same stage, **1:** significant differences compared to stage I at the same tissue, **2:** significant differences compared to stage II at the same tissue, **3:** significant differences compared to stage III at the same tissue (Student’s *t*-test; *p* < 0.05). Original blots are presented in Supplementary Information File S1.

**Figure 4:**
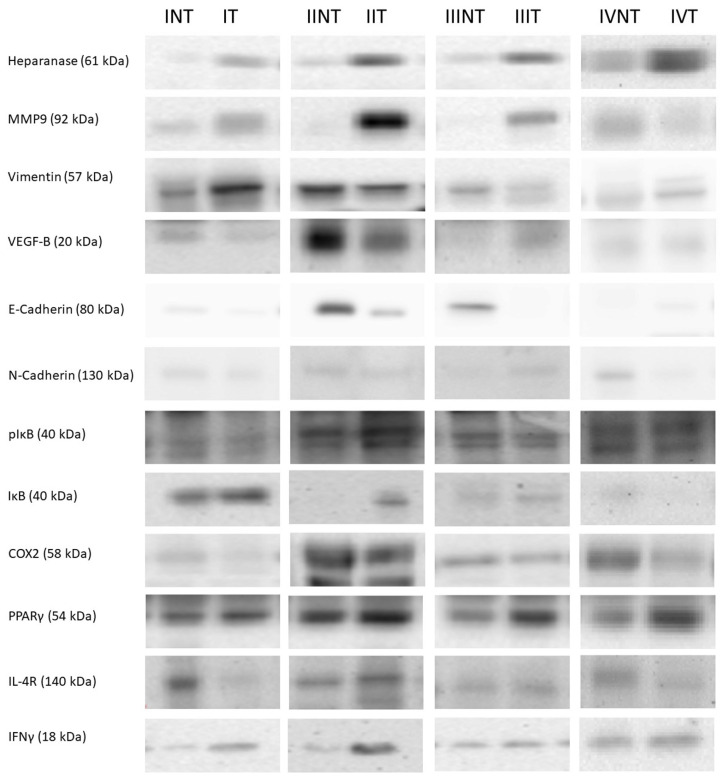
Western blot representative bands and their molecular weight. **NT:** non-tumor adjacent tissue, **T:** tumor tissue, **I:** stage I, **II:** stage II, **III:** stage III, **IV:** stage IV. The whole Western blots were shown in File S1.

pIκB, IκB, pIκB/IκB ratio, COX2, PPARγ, IL-4R and IFNγ protein levels were studied in both tumor and non-tumor adjacent tissues in all stages from CRC patients (see Figure 2, Figure 3 and Figure 4).

pIκB levels (Figure 2A) presented differences between stages, being stage I pIκB levels lower than stages II and III in both tissues and lower than stage IV in non-tumor adjacent tissue. IκB levels (Figure 2B) also showed differences between stages, and an interactive effect between tissue and stage. Stage I IκB levels were higher in both tissues, and non-tumor adjacent tissue stage III presented higher IκB levels than stages II and IV from the same tissue. Furthermore, pIκB/IκB ratio was also studied to estimate the IκB degradation and the NFκB released to the nucleus. pIκB/IκB ratio (Figure 2C) presented differences between stages and an interactive effect between tissue and stage. Non-tumor adjacent tissue stage II showed an increase in the ratio compared to tumor tissue stage II. Moreover, this tissue presented higher levels than non-tumor adjacent tissue stages I and III. In addition, non-tumor adjacent tissue stage IV presented an increase in the ratio compared to stage I. Finally, tumor tissue stages II, III, and IV presented an increase in the ratio levels comparing to stage I, being stage IV levels the highest.

COX2 levels (Figure 3A) presented differences between non-tumor adjacent tissue and tumor tissue, being non-tumor adjacent tissue COX2 levels higher than tumor tissue.

PPARγ levels (Figure 3B) showed differences between non-tumor adjacent tissue and tumor tissue, being tumor tissue PPARγ levels higher than non-tumor adjacent tissue.

IL-4R levels (Figure 3C) presented differences between stages in the same tissue and an interactive effect between tissue and stage. Non-tumor adjacent tissue stages II, III, and IV, showed a decrease in IL-4R levels when compared to stage I. Additionally, stage III also presented lower levels than stage II. Furthermore, tumor tissue stage II showed an increase in IL-4R levels compared to tumor tissue stages I and III. Finally, tumor tissue stage I presented lower IL-4R levels than non-tumor adjacent tissue stage I.

IFNγ levels (Figure 3D) presented a tissue effect, being tumor tissue IFNγ levels higher than non-tumor adjacent tissue.

### 3.3. Vimentin and VEGF-B Expression Levels Are Related to Relapse-Free Survival of Colorectal Cancer Patients in Stage II

**Figure 5:**
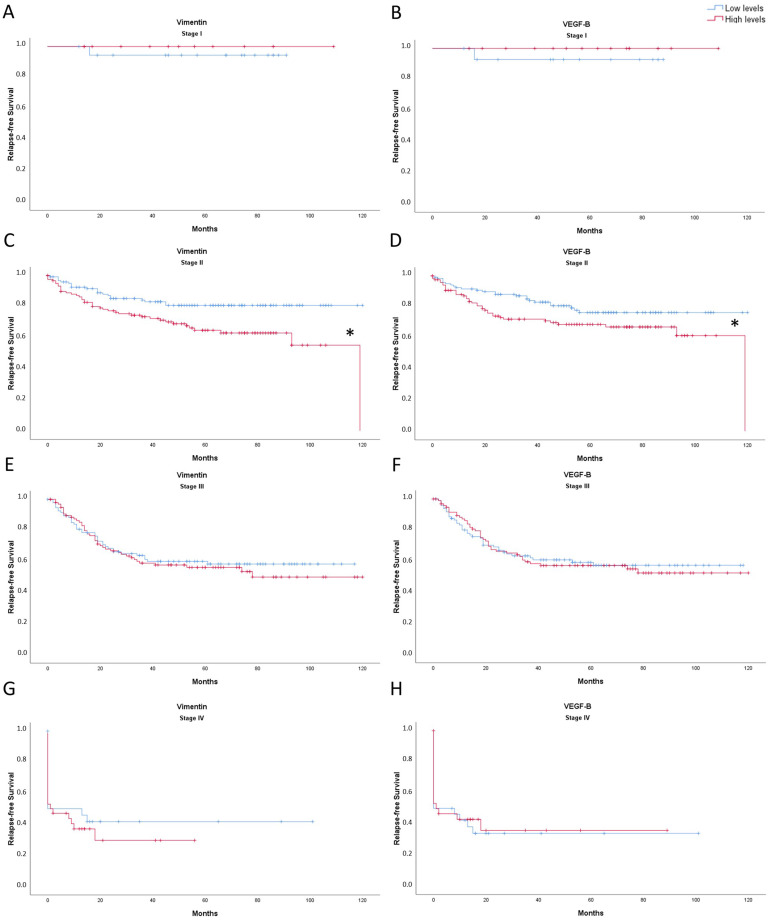
Kaplan-Meier representation of relapse-free survival of Vimentin: ((**A**): stage I; (**C**): stage II; (**E**): stage III; (**G**): stage IV); and VEGF-B ((**B**): stage I; (**D**): stage II; (**F**): stage III; (**H**): stage IV) expression levels of CRC patients for 10 years. The blue line indicates low expression levels and red line indicates high expression levels. * Significant differences between low expression levels and high expression levels (Log Rank; *p* < 0.05).

Vimentin and VEGF-B expression levels were studied according to the tumor stage (I, II, III, and IV) to link them to relapse-free survival of CRC patients (Figure 5). For stage II patients, the higher the expression levels of both vimentin (Figure 5A) and VEGF-B (Figure 5B), the lower 10-year relapse-free survival. No statistically significant results were found in CRC patients in stages I, III, and IV.

## 4. Discussion

**Figure 6:**
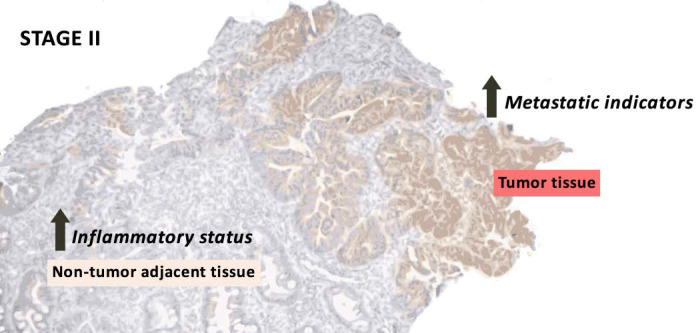
Summary of the most relevant results of stage II in tumor and non-tumor adjacent tissue.

Changes in proteins related to metastasis and inflammation were studied through all stages (stages I, II, III, and IV) in tumor tissue and non-tumor adjacent tissue from colorectal cancer patient’s biopsies. In summary, stage II tumor tissue presented an increase in heparanase and IL-4R, while stage II non-tumor adjacent tissue presented an increase in VEGF-B and the pIκB/IκB ratio. In addition, tumor tissue showed an increase in heparanase, MMP9, PPARγ, and IFNγ, while non-tumor adjacent tissue showed an increase in E-cadherin and COX2 and a decrease in IL-4R. Finally, high expression of vimentin and VEGF-B in stage II are associated with a lower 10-year relapse-free survival. Briefly, the results obtained could indicate an increase in metastatic indicators in early stages, concretely stage II. As expected, this was observed only in tumoral tissue, but non-tumor adjacent tissue showed an increase in inflammatory markers, also in early stages (Figure 6).

The results obtained in tumor tissue could indicate that metastasis-signaling pathways could be initially stimulated in stage II, since our results showed a huge increase in heparanase levels in tumor tissue compared to non-tumor adjacent tissue, especially in stage II. This is in line with Friedmann et al. results, who demonstrated that heparanase was expressed at early stages of neoplasia, but was non-detectable in normal mucosa [16]. Heparanase’s main function is to degrade heparan sulfate chains in the extracellular membrane. Thus, heparanase facilitates several processes including extracellular membrane dissociation, tissular remodeling, cellular migration and dissemination, angiogenesis, metastasis, and inflammation, releasing growth factors and cytokines. Heparanase also regulates inflammation-related genes transcription and inflammatory cell extravasation, and accelerates primary tumor growth [17,18]. Furthermore, our results show that heparanase can be found elevated in non-tumor adjacent tissue in a stage-dependent manner. This could indicate that heparanase is exerting a proinflammatory role [17], which could mean that non-tumor adjacent tissue also presented an increase in its inflammatory status that could be the basis for future tumor initiation.

It has been shown that heparanase can also regulate MMP9 transcription [18]. The main function of this metalloproteinase is to degrade collagen IV from the basement membrane, and it is related to tumoral invasion, metastasis, and angiogenesis induction [19]. MMP9 results showed a similar pattern to non-metastatic stages (I, II, and III) in tumor tissue heparanase levels, as MMP9 presented a huge increase in tumor tissue compared to non-tumor adjacent tissue, especially in stage II. Other authors have also shown overexpression of MMP9 in tumor tissue [20,21]. Following the pattern observed in heparanase and MMP9 levels, vimentin showed a tendency to decrease in stage III when compared to stage II in tumor tissue. Vimentin gene expression has been found to decrease in high-grade CRC tumors [22], while Zhao et al. demonstrated in hepatocellular carcinoma that there were no differences between tumor and non-tumor tissue [23]. Vimentin is considered an EMT-biomarker [23] that participates in the regulation of different physiological functions, such as maintaining cell shape and cytoplasm integrity, attachment, migration, and cellular signalling [22]. The results obtained from datasets, which indicate that an increase of vimentin expression levels promotes a lower relapse-free survival in stage II, could also support our results indicating that vimentin at stage II could be important for the establishment of metastasis. Considering the results of heparanase, MMP9, and vimentin together, the first signs of EMT could occur in stage II.

In addition, E-cadherin and N-cadherin are two proteins involved in EMT, being E-cadherin an epithelial biomarker of EMT and N-cadherin a mesenchymal biomarker of EMT [24]. Downregulation of E-cadherin increases low differentiation, distant metastasis, vascular invasion, lymph node metastasis, lymphatic invasion, and infiltration [25], while the overexpression of N-cadherin is implicated in tumor differentiation, size and lymph node infiltration, and metastasis [24]. Our results showed an increase in E-cadherin in non-tumor adjacent tissue and in tumor tissue stage II, accompanied by no changes in N-cadherin, which has always opposite expression levels. This suggests that the high expression of E-cadherin could be maintaining cells in the epithelial phenotype and the drop of its expression in stage IV could trigger the mesenchymal phenotype transformation and the metastatic process.

As seen for the other proteins, VEGF-B levels were also increased in stage II in tumor tissue. The main function of VEGF-B is to participate in angiogenesis, stimulating the proliferation of endothelial cells and vessel growth. In addition, VEGF-B is also involved in the maintenance of blood vessels, avoiding cell death and initiating tumorigenesis [26,27,28]. For these reasons, the increase observed in tumor tissue stage II could suggest that VEGF-B would be participating in the preservation of blood vessels and avoiding cell death to sustain tumor growth. Considering that VEGF-B levels also presented an increase in stage II non-tumor adjacent tissue, VEGF-B could additionally be related to future tumor initiation [28], in line with our observations of the first signs of metastasis in this stage. In addition, the results obtained from datasets, which indicate that an increase of VEGF-B expression levels promotes a lower relapse-free survival in stage II, could also support that metastasis signalling could be starting in stage II. No difference between tumor and non-tumor adjacent tissue was observed, similar to what was reported for gastro-oesophageal cancer and colon adenocarcinoma [26,29].

Accompanying the increase in stage II in tumor tissue of EMT and metastasis-related proteins, there was also an increase in the inflammatory status in both tumor tissue and non-tumor adjacent tissue, caused by the increase in pro-inflammatory-related proteins and the decrease in anti-inflammatory-related proteins. The increase in metastasis-related proteins in stage II on tumor tissue could be achieved by an increase in inflammation status, since inflammation participates in each colon cancer phase [4]. On the other hand, the increase in inflammatory status in non-tumor adjacent tissue could be the basis for future tumor initiation since inflammation in the tumoral microenvironment is associated with tumoral growth, angiogenesis, EMT, and metastasis [4].

In addition to heparanase and VEGF-B results previously explained in non-tumor adjacent tissue, the IκB ratio presented the higher levels at stage II in non-tumor adjacent tissue. This increase can be translated into an increase in inflammation, as IκB is a protein that inhibits nuclear factor kappa B (NF-κB) and, when IκB is phosphorylated by IκB kinase (IKK), IκB is degraded by proteosome and NF-κB is released [30]. NF-κB is a transcription factor that, when heterodimerizes, can be translocated into the nucleus, where it induces the transcription of genes related to inflammation, cell growth, and the immune response [30]. This increase in inflammation could mean that non-tumor adjacent tissue in stage II patients is initiating and promoting an inflammatory status that can support tumoral growth. Furthermore, the IκB ratio presented an increase in stages II, III, and IV in tumor tissue regarding stage I, which means that there was an increase in inflammatory status [30].

Accordingly to the IκB ratio levels, COX2 levels, which can be regulated by NF-κB [30], were higher in non-tumor adjacent tissue. This could contribute to perpetuating the inflammatory status in this tissue, as well as increasing cell proliferation, since COX2 converts free arachidonic acid into prostaglandins to stimulate inflammation, angiogenesis, and tumoral growth [31,32]. Furthermore, it has been seen that COX2 can increase proliferation and invasiveness in epithelial human colon cancer cells [32]. In addition, COX2 levels in non-metastatic stages in tumor tissue also followed the same pattern as the IκB ratio levels, which could also indicate an increase in inflammation at these stages in addition to an increase in angiogenesis, tumoral growth, proliferation, and invasiveness [31,32]. Moreover, heparanase levels also increased in stage II in tumor tissue contributing to the perpetuation of the inflammatory status besides its role in metastasis [17,18].

Furthermore, IFNγ also presented an increase in tumor tissue that was more pronounced in stage II. IFNγ reduces the number of endothelial cells and induces blood vessel destruction and tumor necrosis [33]. Thus, it is possible that IFNγ is elevated in order to palliate the VEGF-B effects by reducing the number of endothelial cells and blood vessels and producing necrosis. In this regard, in stage III, IFNγ could be reduced to avoid necrosis and continue with metastasis.

On the other hand, our results suggest that the anti-inflammatory proteins cannot palliate the inflammatory status increase. IL-4R presented a decrease in all three stages II, III, and IV in non-tumor adjacent tissue regarding stage I. This decrease could mean that IL-4 cannot alleviate the promotion of inflammation started in non-tumor adjacent tissue because IL-4 has an anti-inflammatory role, as it downregulates NF-κB transactivation induced by proinflammatory cytokines, decreasing IκB proteasome degradation [34]. In tumor tissue, IL-4R presented a decrease in tumor tissue, which could mean that IL-4 cannot palliate the inflammatory status present in tumor tissue, thus perpetuating it. Finally, PPARγ has anti-inflammatory properties since it inhibits inflammation-related genes [35] and can inhibit NF-κB in different ways [34,36,37]. PPARγ levels presented an increase in tumor tissue, which was also seen by DuBois et al. [38], that can be interpreted as an insufficient attempt of PPARγ to avoid IκB phosphorylation for maintaining NF-κB at the cytoplasm and decrease the inflammatory status.

Further studies are necessary for a better understanding of metastasis initiation and the associated inflammation. An interesting approach could be the development of 3D models in vitro from a primary explant of CRC tissues to understand in a better way the EMT and metastasis processes. Several approaches can be used to mimic the in vivo cancer environment, including the use of biomaterials [39] or even the use of 3D printed tissue constructs [40], and 3D bone-on-a-chip systems that can allow the study of the bone metastasis [41]. Unfortunately, there are no animal models for the different CRC stages (I, II, III, IV) since there are only animal models for early or late CRC. This situation makes it difficult to investigate the different markers in vivo and across the different stages, despite the existence of some advanced studies about the use of zebrafish xenografts and *Drosophila melanogaster* [42,43].

## 5. Conclusions

In conclusion, these results could suggest that some metastasis-related signalling pathways may be activated in stage II in tumor tissue due to an increase in inflammation at this stage. Furthermore, non-tumor adjacent tissue presented an increase of the inflammatory status that could be the basis for future tumor progression. Although further analyses are necessary to better understand the metastasis initiation in colorectal cancer and the inflammation associated with the metastatic process, this study indicates the convenience of determining the possibility of using the proteins measured as biomarkers of diagnosis and evolution in the early stages of colon cancer.

## Data Availability

The data presented in this study are available in this article and supplementary material.

## Supplementary Materials

The following supporting information can be downloaded at: https://www.mdpi.com/article/10.3390/cancers14184487/s1, Table S1: clinicopathological features of patient’s samples included in the study, File S1: The whole Western blots.
