# Supplementary material for: Inflammation- and Metastasis-Related Proteins Expression Changes in Early Stages in Tumor and Non-Tumor Adjacent Tissues of Colorectal Cancer Samples
Source: Cancers (Basel). 2022 Sep 16;14(18):4487. doi: 10.3390/cancers14184487 (PMC9497293; doi:10.3390/cancers14184487)
Supplement: Supplementary file 1 [file cancers-14-04487-s001.zip › cancers-1876517-supplementary.pdf]

Supplementary Table S1. Clinicopathological features of patient's samples included in the study.

Age	BMI	Stage	TNM	Histological grade	Tumor location	Metastasis
78	25.6	I	T2N0M0	-	-	No
56	-	I	T1N0MX	G2	Sigmoid colon	No
59	-	I	T2N0MX	G1	Sigmoid colon and rectum	No
83	28.2	I	T2N0M0	G2	Descending and rectum	Yes (local and lung)
79	-	I	T2N0MX	G2	Descending	No
65	-	I	T2N0M0	G3	Ascending	No
64	-	I	T2N0MX	G2	Descending	No
56	-	I	T2PN0M0	G1	Descending	No
77	25.5	IIA	T3BN0MX	G3	Right colon at hepatic angle	No
78	29.7	IIA	T3N0MX	G2	Rectum - sigmoid union	No
70	22.6	IIA	PT3BN0M0	G2	Rectum	No
67	26.7	IIA	T3CN0M0	G2	Rectum – sigmoid union	No
60	-	IIA	T3(M)N0MX	G1	Ascending	No
74	-	IIA	T3BN0MX	G3	Ascending	No
72	-	IIA	T33N0MX	G1	Descending	No
50	-	IIA	T3CPN0M0	G2	Descending	No
88	-	IIA	T3N0M0	G1	Sigmoid	No
81	20.8	IIA	T3PN0M0	G2	Descending	No
81	24.2	IIIB	T3BN1M0	G1	Transverse	No
82	26.3	IIIB	T3N1M0	G2	Ascending – Caecum	No
50	25.5	IIIC	T4BPN2M0	G2	Sigmoid	No
67	24.9	IIIC	T3N2BM0	G3	Ascending	No
70	22.1	IIIA	T2N1M0	G2	Rectum	No
51	22.6	IIIC	T4BN2M0	G2	Sigmoid	No
69	27.6	IIIB	T3N1M0	G3	Ascending	No
83	24.7	IIIB	T3N2M0	G3	Ascending	No
92	-	IVA-B	T3N2M1	G2	Transverse - Sigmoid	Yes
83	-	IVA-B	T3BN2MX	G3	Ascending - Caecum	Yes
63	24.4	IVA-B	T2PN2PM1	-	Descending - Rectum	Yes
78	33.5	IVA-B	T3PN1M1	G1	Hepatic angle	Yes
52	23.4	IVA-B	T3N1M1	-	Sigmoid	Yes
80	23.6	IVA-B	T4N2M1	G2	Sigmoid	Yes
55	20.3	IV-B	T4PN2M1	G3	Ascending	Yes
76	23.4	IVA-B	T3N1M1	G2	Ascending	Yes
67	34.2	IVA-B	T3PN2M1	G3	Ascending	Yes
74	18.8	IVA-B	PT4PN0M	G3	Rectum	Yes
65	32.9	IV	T3N2M1	G2	Sigmoid - Rectum	Yes
95	27.9	IV	T3N2M1	G2	Ascending - Transverse	Yes
