# Supplementary material for: Inflammation- and Metastasis-Related Proteins Expression Changes in Early Stages in Tumor and Non-Tumor Adjacent Tissues of Colorectal Cancer Samples
Source: Cancers (Basel). 2022 Sep 16;14(18):4487. doi: 10.3390/cancers14184487 (PMC9497293; doi:10.3390/cancers14184487)

Supplementary information File S**1**: Original blots from Western blot analysis with the corresponding figure of the main text in bold, loading order at each band and density of each band before the image. **Mk** molecular weight marker; **x** non-related sample; **NT** non-tumor adjacent tissue; **T** tumor tissue; **1** stage I; **2** stage II; **3** stage III.

Heparanase (Figure S1A)

Mk/222/1012/1385/468/2739/82/1196/2876/54/55/189/3781/x/mk

56/72/mk/1894/83/78/77/284/2144/58/208/92/1241/x/mk

46/1580/38/1413/mk/5628/42/1044/411/1773/3629/x/3619/x/mk

73/923/112/x/76/967/mk/1140/131/82/87/77/1966/x/mk

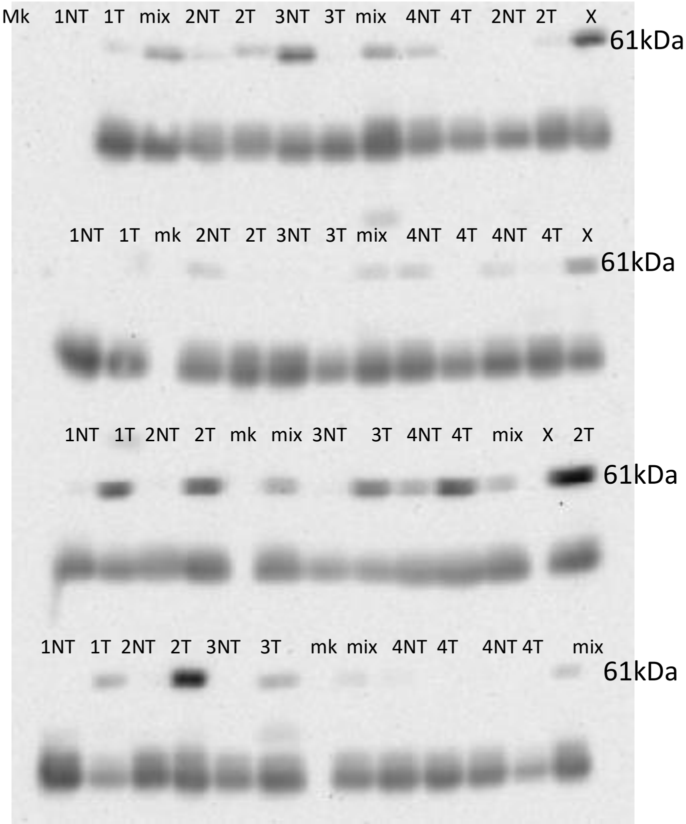

Mk/x/1704/1768/295/4193/327/x/2454/x/1461/112/24/x/mk

29/21/mk/1276/171/2740/142/713/1995/272/28/343/26/x/mk

51/488/x/1803/mk/5732/252/x/159/283/1933/x/mk/x/x

1415/219/x/83/x/315/435/mk/1707/217/2002/x/mk/x/x

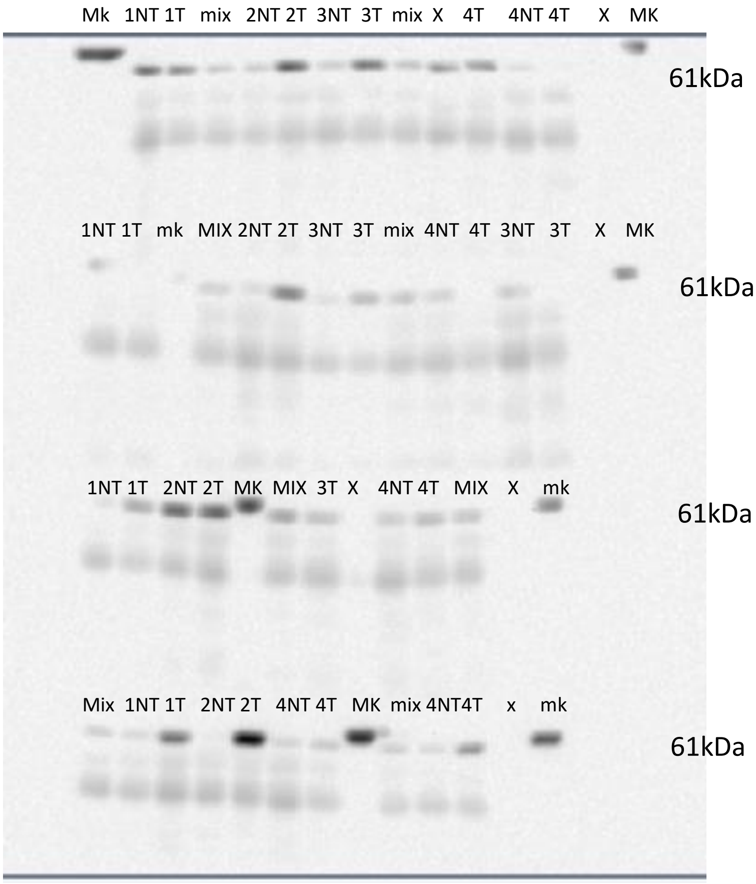

MMP9 (Figure S1B)

Mk/126/33/3795/12/823/53/32/6094/20/25/65/46/x/mk

156/83/mk/5280/158/68/36/40/5399/61/24/x/43/x/mk

58/25/113/222/mk/5217/35/x/109/22/4185/x/mk

4783/58/144/143/183/127/13/mk/1218/20/58/x/mk

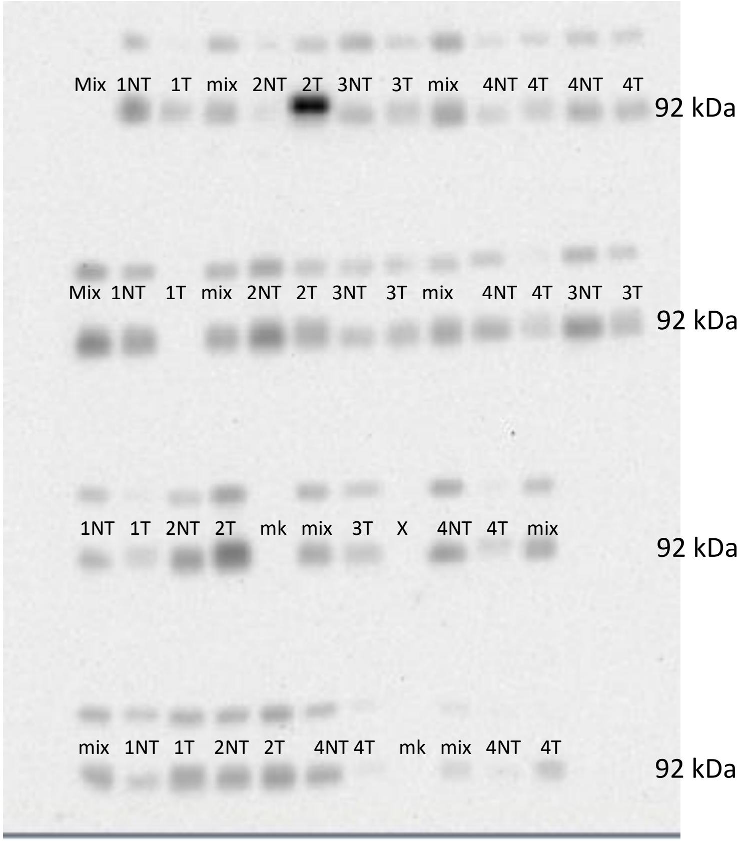

Mk/150/624/4185/162/3481/71/2322/5999/45/113/226/1736/x/mk

83/x/mk/3820/60/142/27/924/4622/35/x/60/91/x/mk

100/x/x/1413/mk/3729/25/1082/171/x/6329/48/x/x/mk

66/731/50/179/43/52/mk/628/104/54/51/45/4479/x/mk

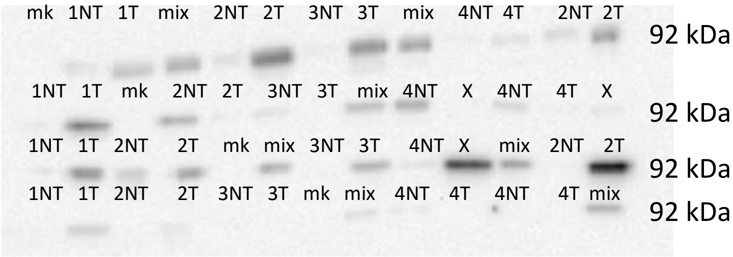

Vimentin (Figure S1C)

Mk/47/x/x/92/520/52/39/66/39/x/x/144/78

36/mk/279/x/1405/136/x/85/51/119/68/68/51/61

122/100/mk/50/x/92/107/867/76/61/x/x/61/57

46/100/x/mk/100/67/x/67/83/67/67/233/933/53

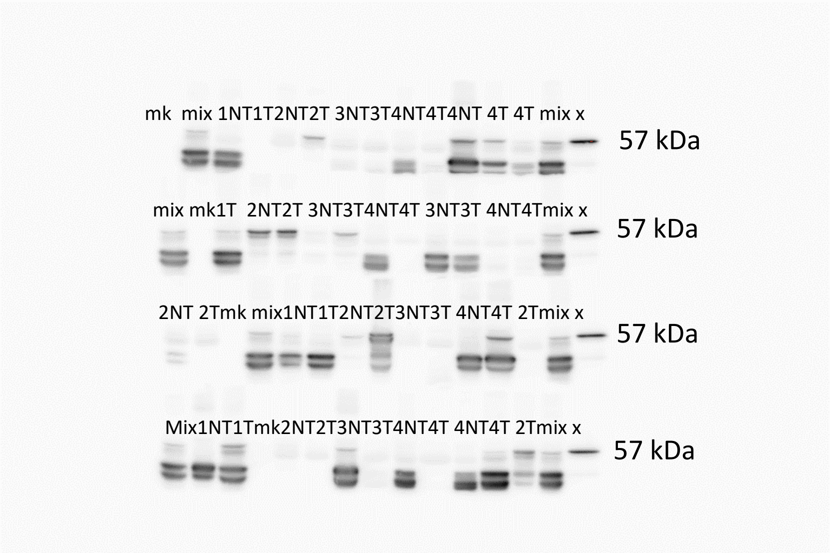

Mk/221/98/94/103/92/61/104/106/94/107/106/107/224

227/mk/106/93/98/96/96/107/103/104/105/107/x/217

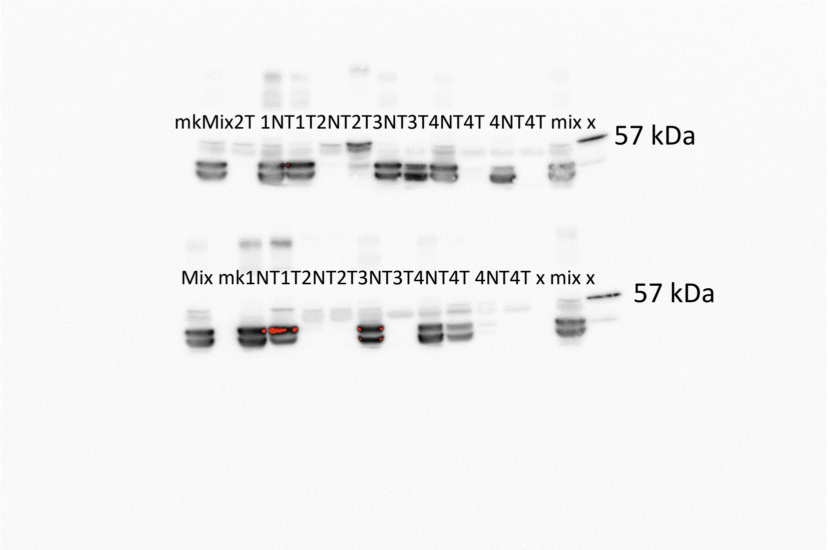

VEGF-B (Figure S1D)

105/mk/63/342/260/62/299/69/48/45/62/65/62/105

190/345/mk/56/84/107/152/634/100/344/154/x/204/68

56/116/70/mk/346/259/118/75/155/x/57/58/227/95

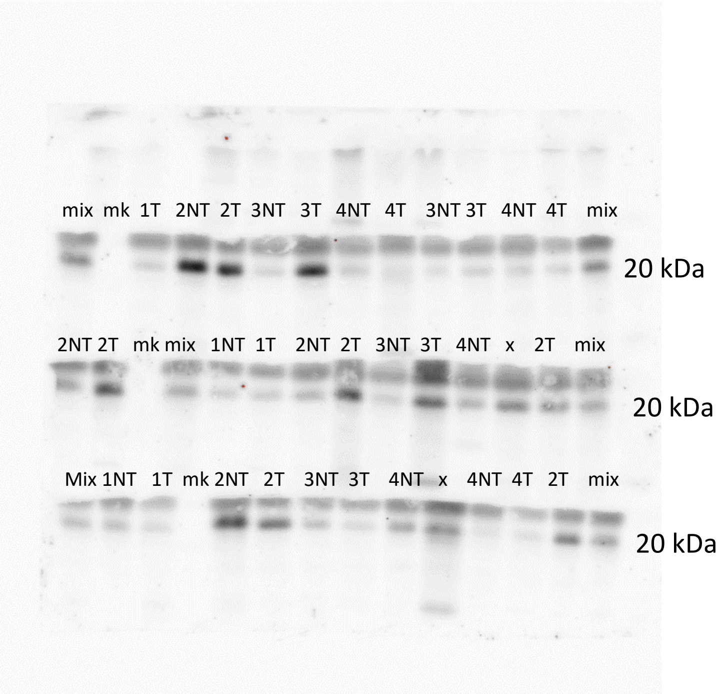

Mk/227/70/98/104/92/78/103/103/104/100/95/97/219

220/mk/102/106/74/76/83/91/99/105/101/106/x/216

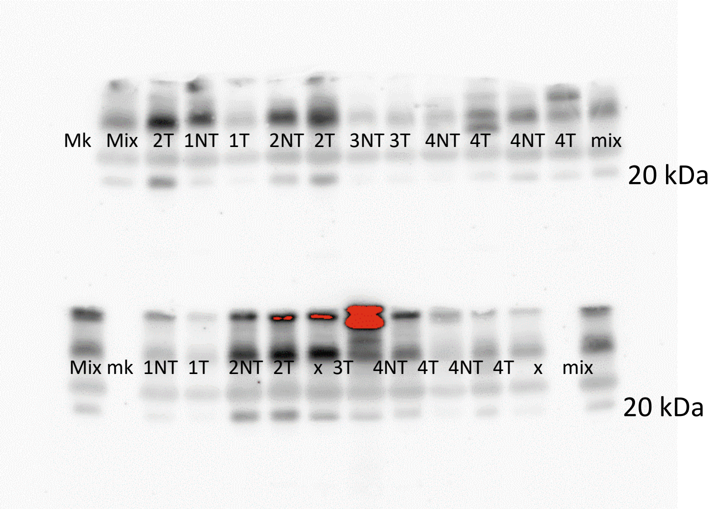

E-Cadherin (Figure S1E)

Mk/54/33/9662/x/17/17/21/17/x/x/25/69

40/mk/95/9990/191/11918/x/29/19/12016/34/19/15/66

11916/x/mk/57/25/215/8770/621/20/25/x/x/466/47

70/242/167/mk/6112/x/10492/15/31/18/19/29/139/70

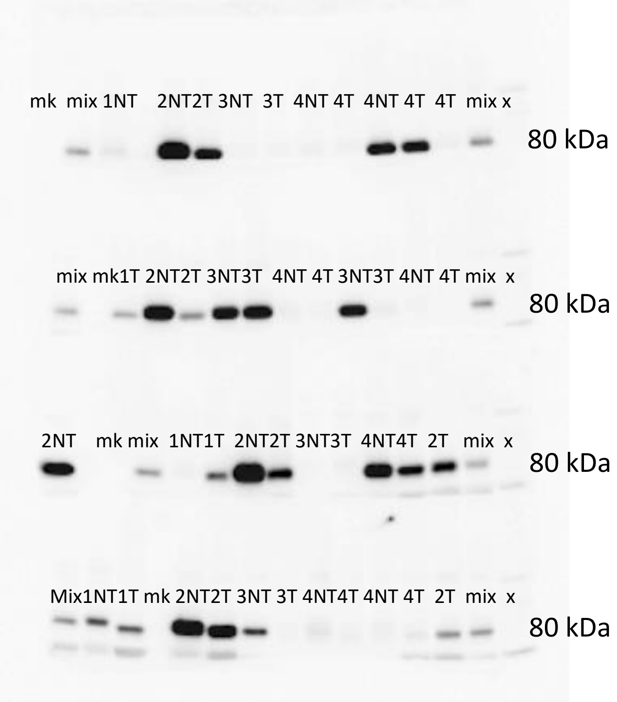

Mk/47/2985/x/109/2482/176/3340/5/6/5/5/5/66

27/mk/100/55/2756/27/123/7/14/9/4220/123/53

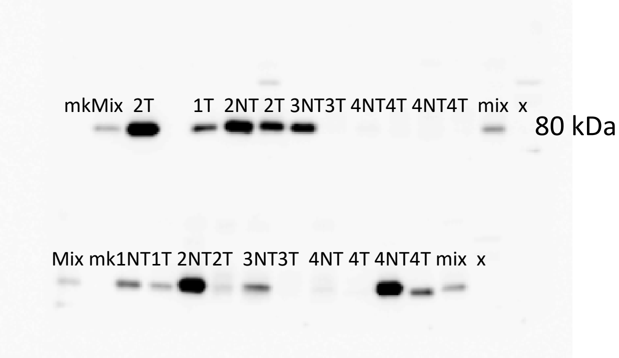

N-Cadherin (Figure S1F)

Mk/34/x/184/121/65/67/82/84/136/55/64/34

35/mk/76/54/102/65/77/79/66/58/62/59/53/29

118/76/mk/32/96/87/62/68/72/99/111/105/58/31

33/104/65/mk/80/74/81/62/146/66/80/73/124/33

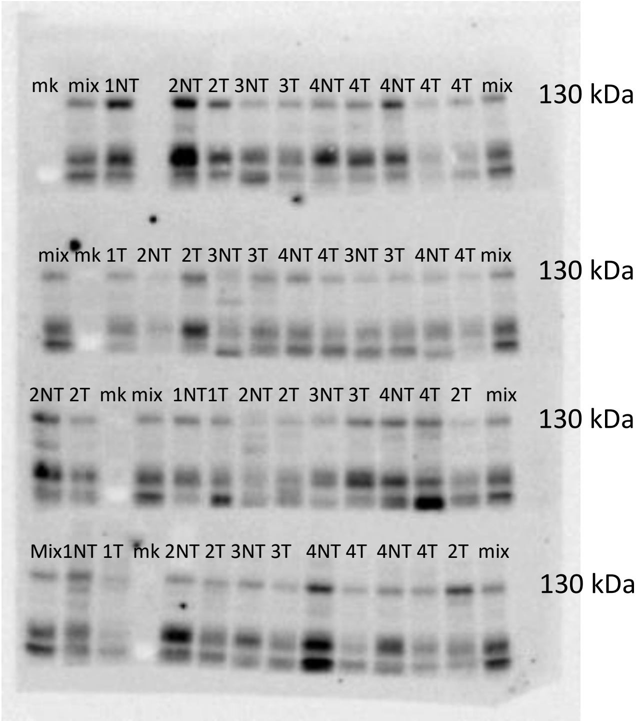

Mk/63/98/95/79/120/104/129/113/121/130/97/61

71/mk/102/91/122/101/131/113/149/98/78/85/55

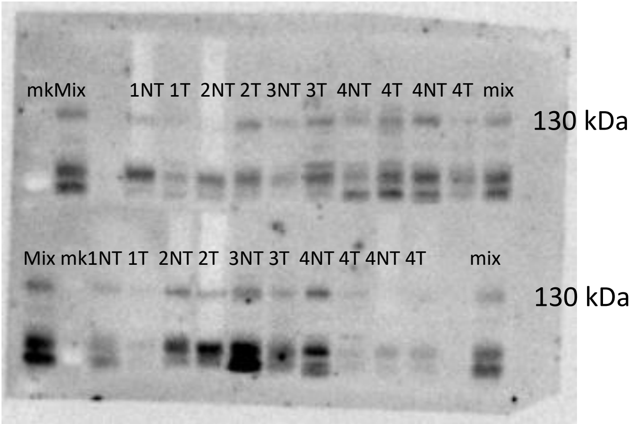

pIκB (Figure S2A)

mk/x/x/x/x/28544/198/201/191/x/x/171/170/x/164/134/110/108/19027/120/103/143/x/mk

128/192/90/x/32792/91/x/mk/x/183/183/196/138/111/88/122/46556/135/170/90/95/x/x/m
k

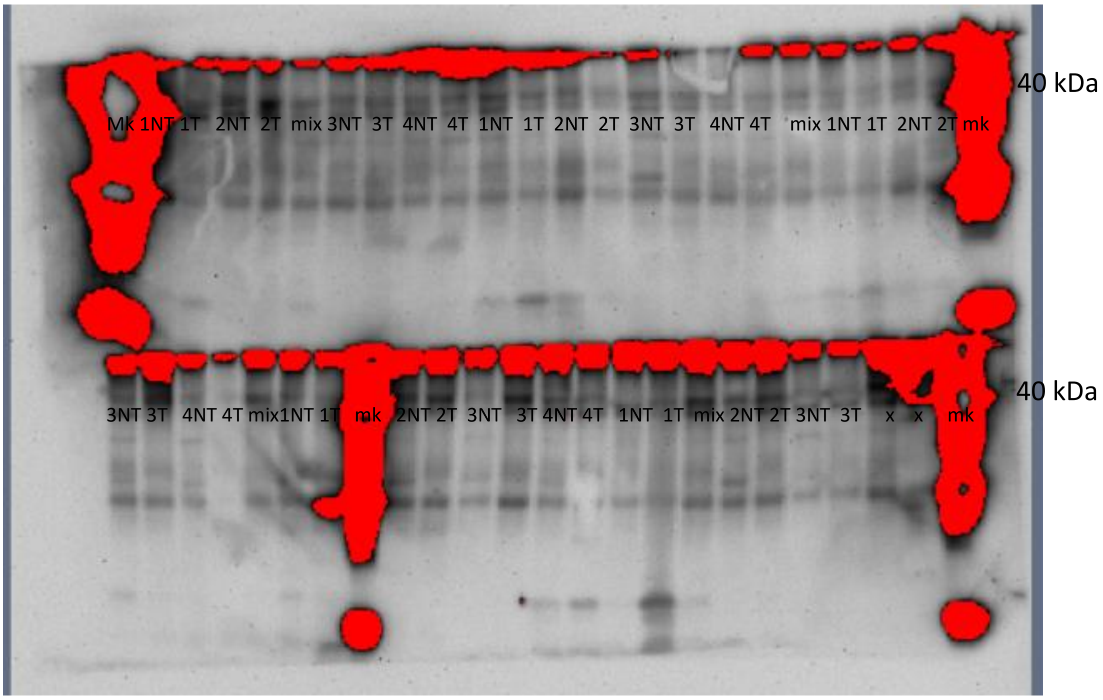

Mk/10300/112/123/107/159/124/109/5825/93/95/85/111/94/112

6392/182/x/150/189/x/x/177/204/179/175/7640/x/x/mk

mk/x/10410/x/155/162/147/9210/128/127/143/137/114/x

10177/148/131/8024/99/88

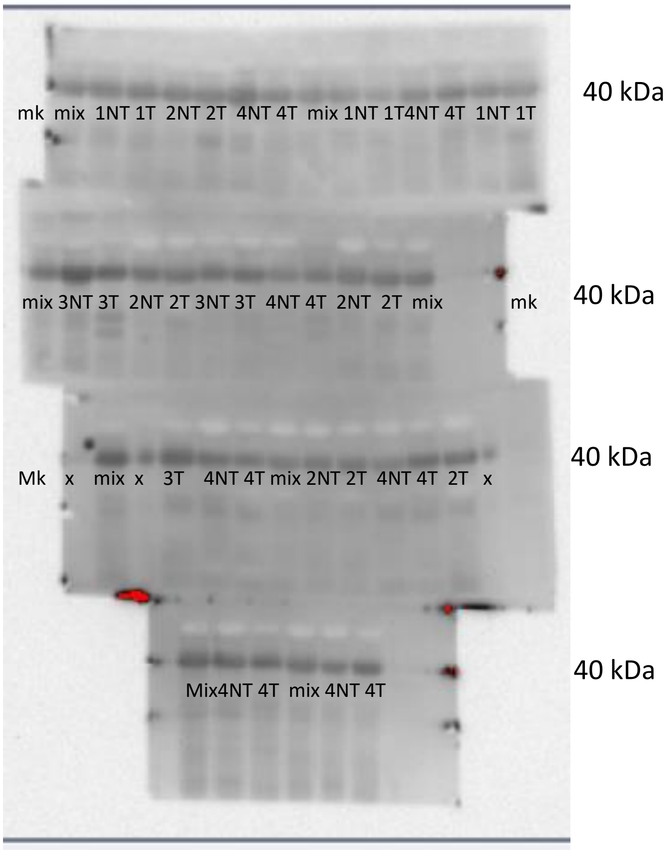

IκB (Figure S2B)

Mk/x/163/48/x/4100/76/73/101/x/x/284/x/72/x/73/49/96/x/107/140/42/x/mk

x/99/75/x/3555/101/x/mk/x/78/54/x/x/92/90/392/7893/102/55/86/55/x/x/mk

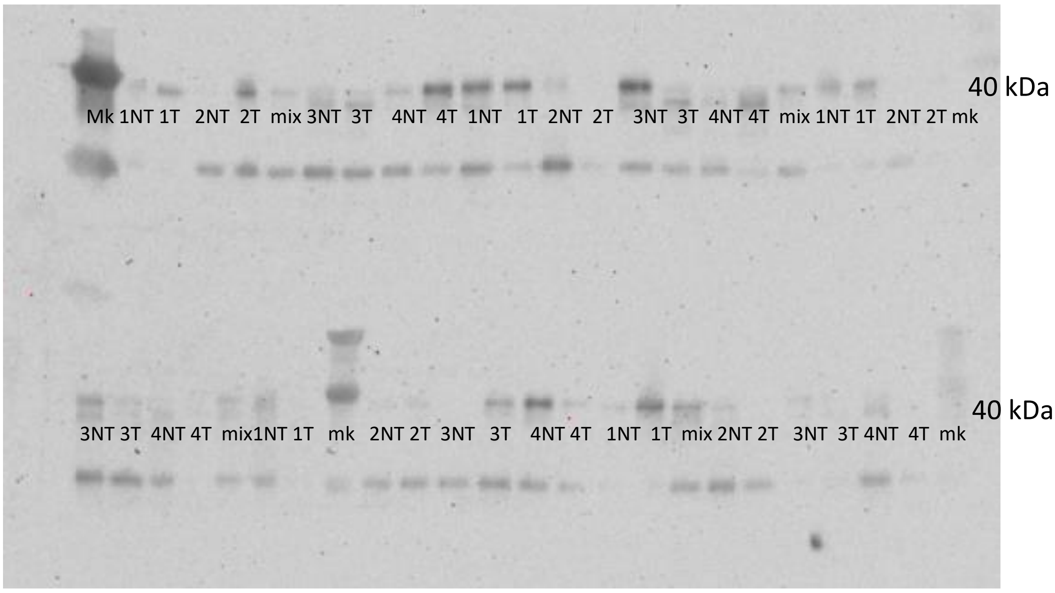

Mk/6283/52/10/13/23/x/x/5752/84/109/x/x/163/249

7472/102/x/14/107/x/x/x/x/10/34/5179/mk

Mk/x/7683/x/31/x/x/5141/9/46/x/x/x/18

7571/13/10/6875/53/9

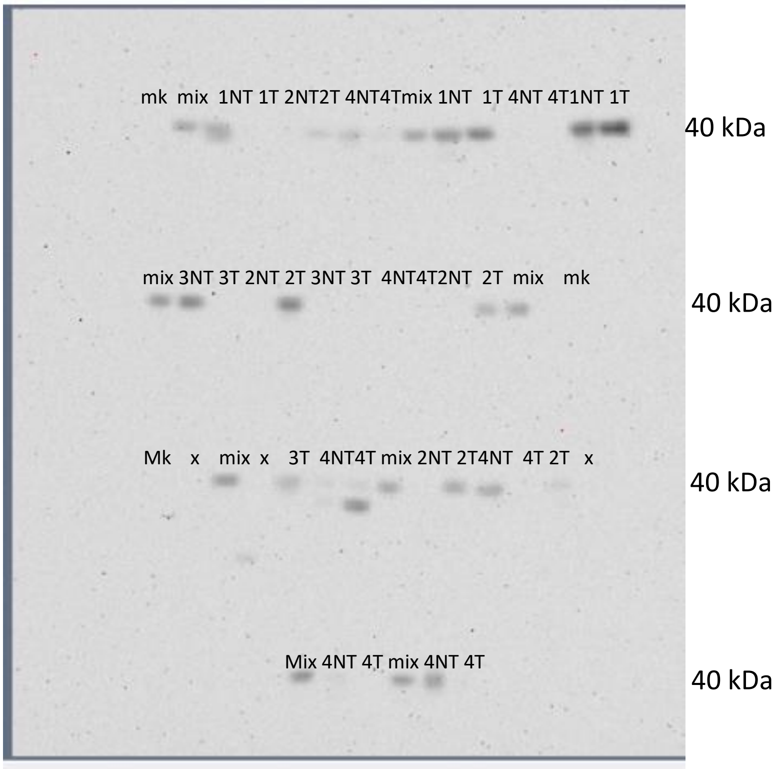

COX2 (Figure S3A)

Mk/100/11/237/152/42487/158/30/111/43/105/15/133/27/205/101/55/19/20507/x/8/109/67/mk

156/40/47/x/21034/105/26/mk/273/182/68/224/112/19/89/15/55341/89/82/29/21/156/20/mk

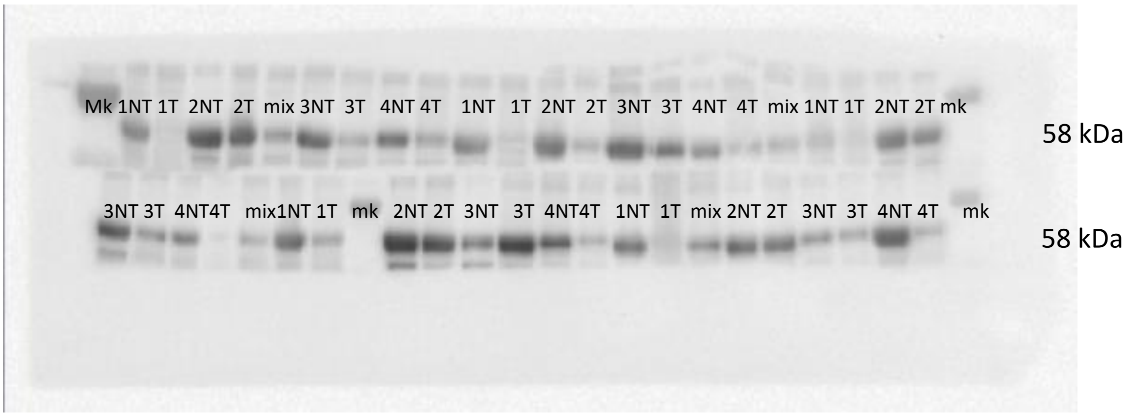

Mk/x/9/115/50/87/94/196/83/5/105/27/274/167/22/134/90/110/31/89/16/349/106/mk

30/22/x/58/160/100/12/83/37/mk/159/31/157/221/210/590/x/105/15/355/32/90/82/mk

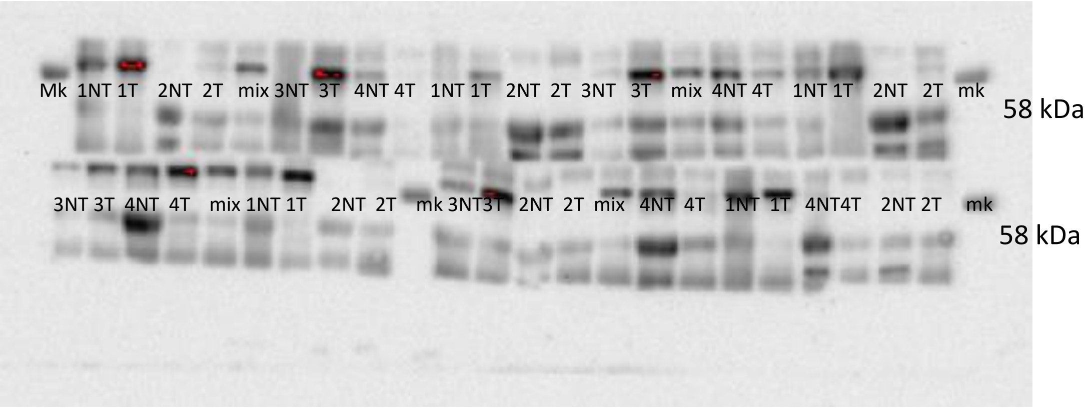

Mk/5534/110/115/157/221/278/90/93/74/6331/144/133/x/mk

3122/x/mk/95/122/291/131/x/x/x/x/x/x/3866/mk

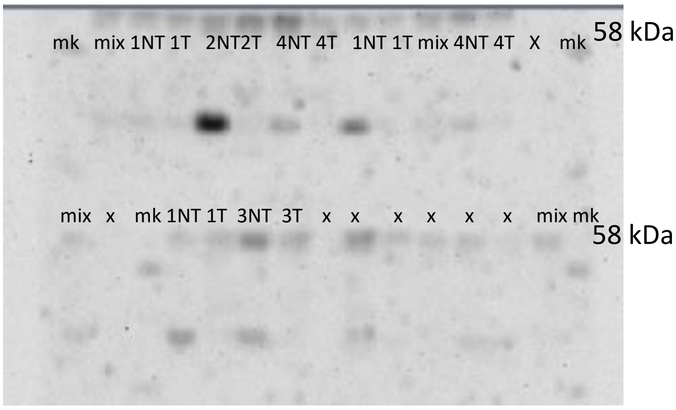

PPARg (Figure S3B)

Mk/6023/118/167/39/518/x/149/23708/116/149/93/97/65/78

5859/mk/200/531/x/x/x/x/x/x/x/x/8545/mk

Mk/3775/x/x/x/x/27054/x/x/142/624/202/x/x/mk

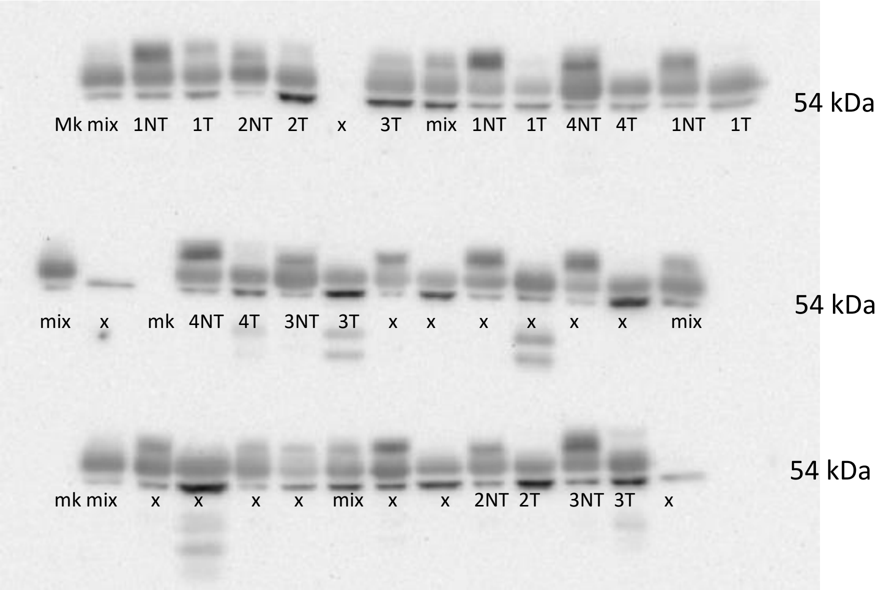

3222/mk/109/164/x/152/143/143/x/459/5226/x/mk

x/6438/mk/195/486/x/480/113/617/95/281/13463/mk

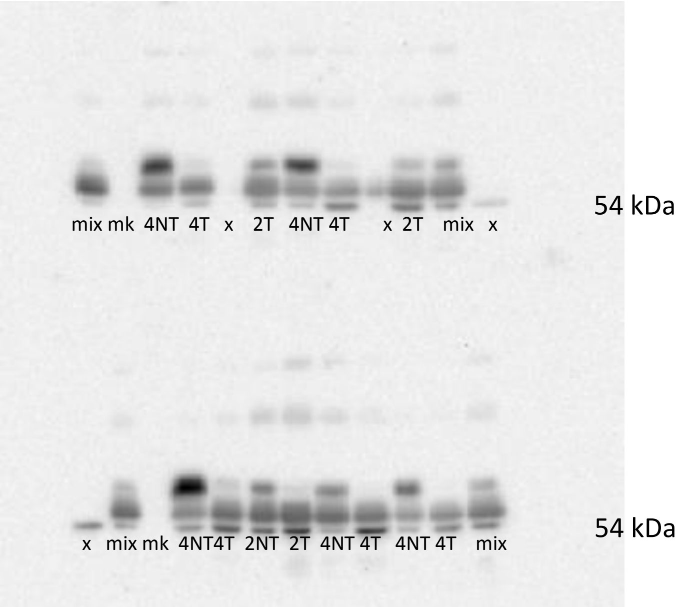

Mk/x/16093/133/328/7519/60/348/61/391/46/265/95/144/mk

10898/x/mk/111/187/138/209/110/573/127/415/68/33/5524/mk

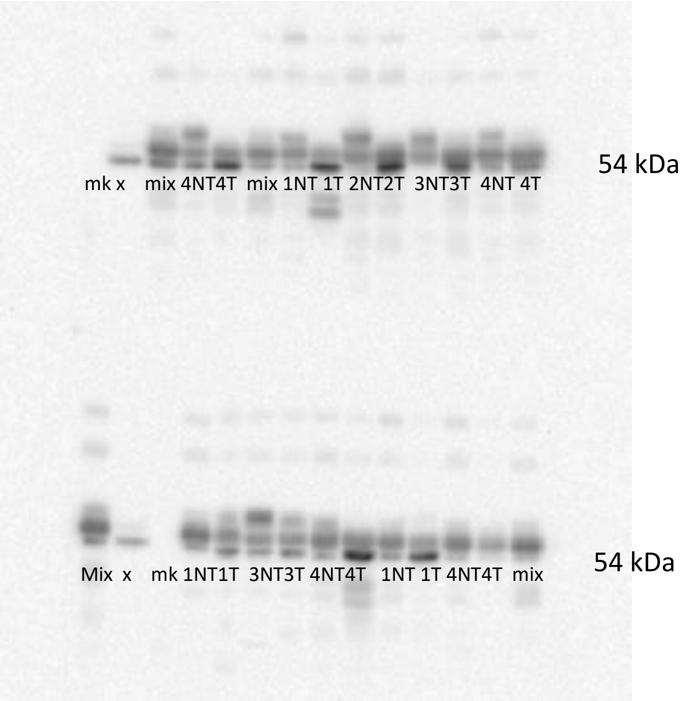

Mk/x/25266/100/126/29354/176/245/21/163/159/175/16/199/mk

28012/x/mk/81/193/2520/104/185/x/96/157/309/142/172/mk

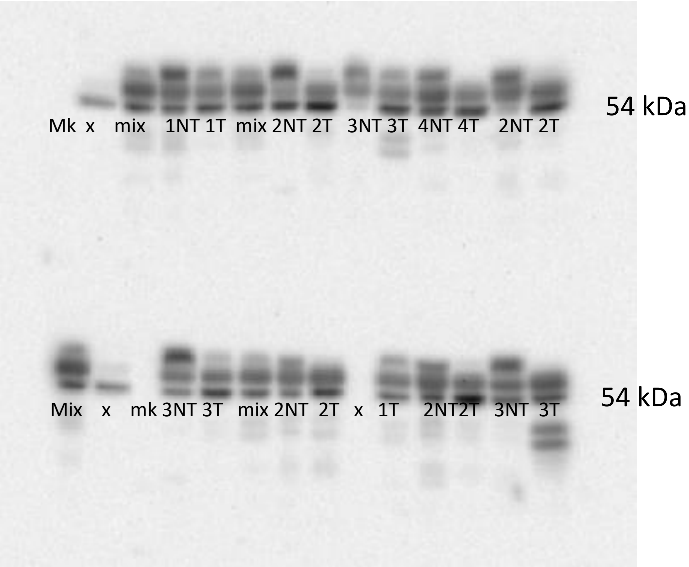

IL-4R (Figure S3C)

Mk/27/42/5049/32/36/67/39/3525/39/23/58/58/x/mk

120/38/mk/3445/36/56/45/41/2985/52/38/x/54/x/mk

121/50/104/x/mk/2723/x/50/x/50/2745/x/mk

3684/130/x/x/178/92/41/mk/2269/39/55/x/mk

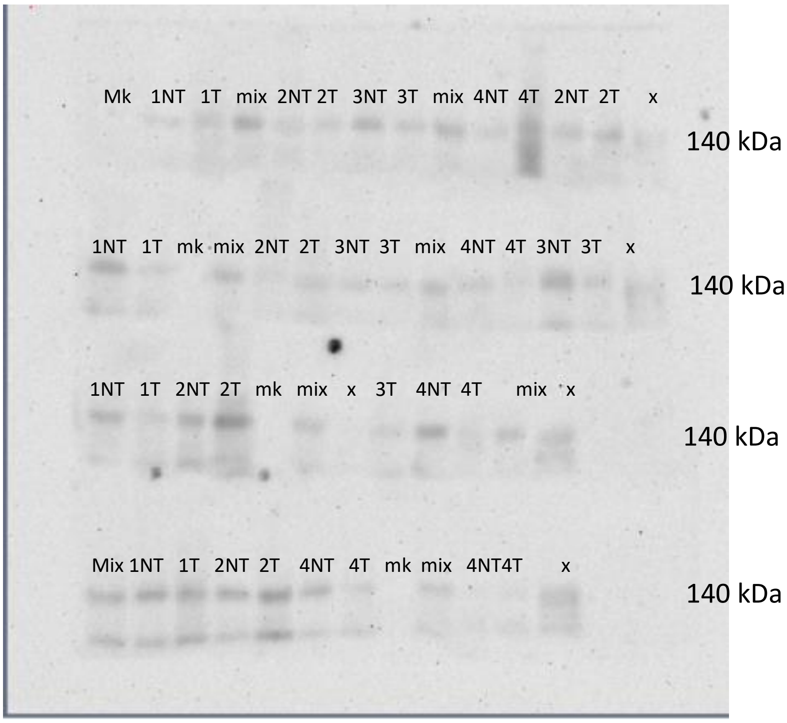

Mk/101/47/11488/75/125/38/21/9183/39/23/58/58/x/mk

163/35/mk/7560/61/71/50/61/9031/38/28/52/46/x/mk

89/61/80/84/mk/7477/39/43/70/84/7701/x/51/x/mk

46/43/72/52/30/77/mk/7690/52/63/83/46/8010/x/mk

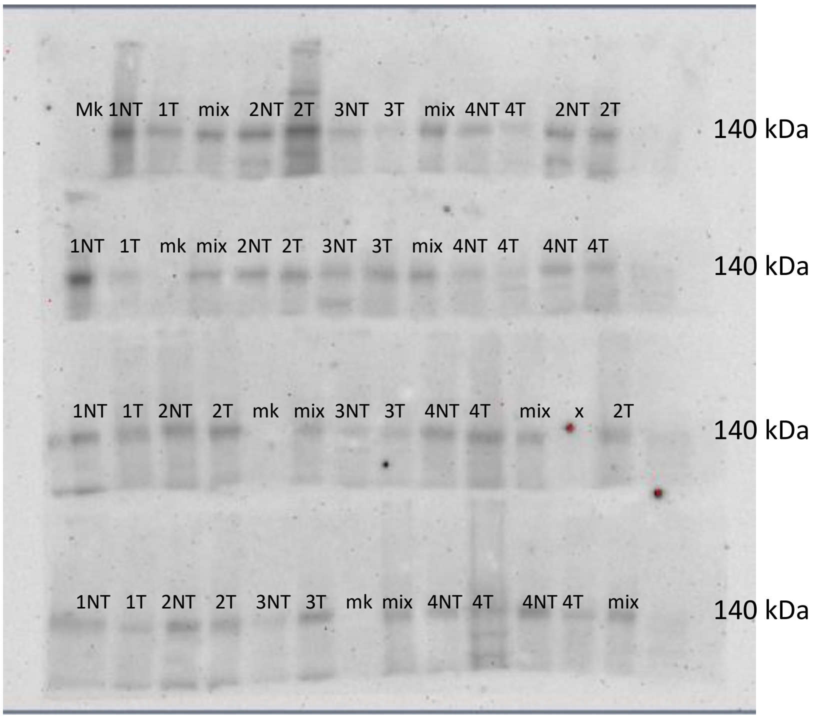

IFNg (Figure S3D)

Mk/6453/152/217/5073/98/96/61/95/69/91/24/97/x/mk

325/x/mk/368/25/x/87/29/85/30/924/113/1728/1835/mk

317/x/146/x/mk/317/190/1826/192/x/232/278/75/x/mk

332/x/256/1265/x/808/mk/598/54/2591/126/157/394/1345/mk

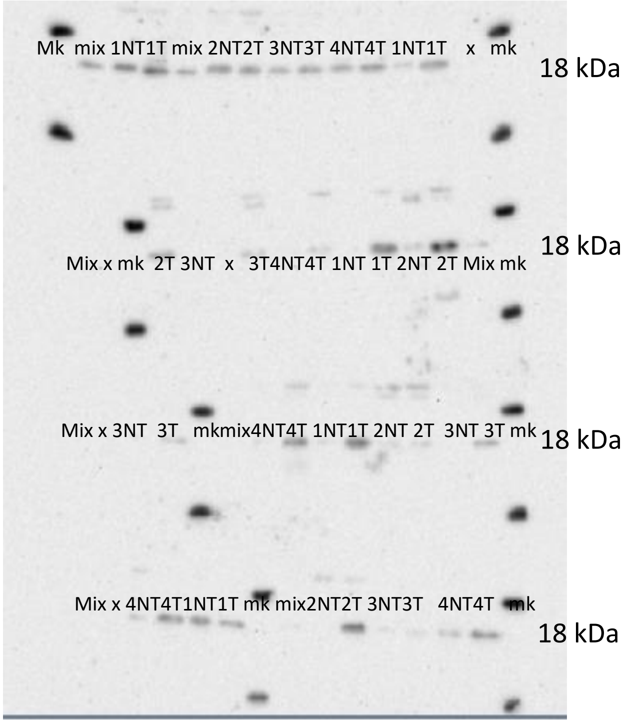

Mk/2159/78/121/250/1179/83/314/95/111/1100/83/75/x/mk

1120/x/mk/126/521/124/156/x/x/118/136/115/114/997/mk

1254/x/194/159/mk/1131/x/100/241/161/198/617/131/133/mk

1192/x/167/196/233/233/1368/mk/220/496

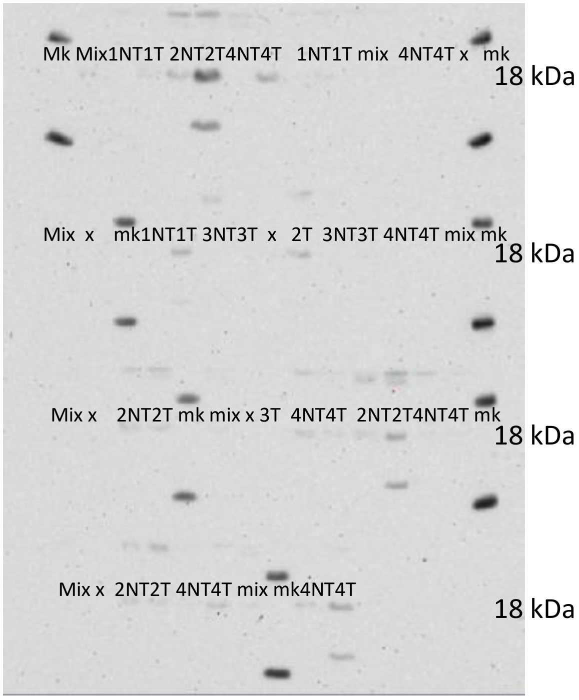

Supplement: Supplementary file 1 [file cancers-14-04487-s001.zip › Supplementary information File S1.pdf]
